# Size-exclusion chromatography small-angle X-ray scattering of water soluble proteins on a laboratory instrument

**DOI:** 10.1107/S1600576718014462

**Published:** 2018-11-09

**Authors:** Saskia Bucciarelli, Søren Roi Midtgaard, Martin Nors Pedersen, Søren Skou, Lise Arleth, Bente Vestergaard

**Affiliations:** a Department of Drug Design and Pharmacology, University of Copenhagen, Denmark; b Structural Biophysics, X-ray and Neutron Science, The Niels Bohr Institute, University of Copenhagen, Denmark; c Xenocs Nordic, Hørsholm, Denmark

**Keywords:** size-exclusion chromatography small-angle X-ray scattering, SEC-SAXS, molecular weight, polydisperse proteins, in-house small-angle X-ray scattering

## Abstract

Coupling of size-exclusion chromatography with biological solution small-angle X-ray scattering (SEC-SAXS) on dedicated synchrotron beamlines enables structural analysis of challenging samples such as labile proteins and low-affinity complexes. For this reason, the approach has gained increased popularity during the past decade. Transportation of perishable samples to synchrotrons might, however, compromise the experiments, and the limited availability of synchrotron beamtime renders iterative sample optimization tedious and lengthy. Here, the successful setup of laboratory-based SEC-SAXS is described in a proof-of-concept study. It is demonstrated that sufficient quality data can be obtained on a laboratory instrument with small sample consumption, comparable to typical synchrotron SEC-SAXS demands. UV/vis measurements directly on the SAXS exposure cell ensure accurate concentration determination, crucial for direct molecular weight determination from the scattering data. The absence of radiation damage implies that the sample can be fractionated and subjected to complementary analysis available at the home institution after SEC-SAXS. Laboratory-based SEC-SAXS opens the field for analysis of biological samples at the home institution, thus increasing productivity of biostructural research. It may further ensure that synchrotron beamtime is used primarily for the most suitable and optimized samples.

## Abstract 2

Size-exclusion chromatography coupled with a laboratory-based small-angle X-ray scattering setup is presented and its performance evaluated for studies of various proteins covering a broad range of molecular weights.

## 1. Introduction   

Solution-based small-angle X-ray scattering (SAXS) has over recent decades gained popularity in structural biology, owing to its potential to investigate the structure, dynamics and interactions of biomolecules directly in solution (Petoukhov & Svergun, 2013 ▸; Bizien *et al.*, 2016 ▸; Vestergaard, 2016 ▸). Such structural insights usually require monodisperse samples devoid of aggregates and impurities, as each individual component of a solution contributes to the total scattering pattern (Jacques & Trewhella, 2010 ▸; Rambo & Tainer, 2013 ▸; Jeffries *et al.*, 2016 ▸; Rambo, 2017 ▸). This requirement can be challenging to meet for biological samples from a sample preparation point of view, not least because a vast number of biological processes, including pathological cases, involve structurally heterogeneous and aggregation-prone multi-domain proteins (Han *et al.*, 2007 ▸) and heterogeneous protein complexes in dynamic equilibria (Ali & Imperiali, 2005 ▸; Berger *et al.*, 2011 ▸; Knowles *et al.*, 2014 ▸; Marsh & Teichmann, 2015 ▸; Vestergaard, 2016 ▸). Structural investigation of such systems is highly relevant, but often obstructed by the structural dispersity and unstable or transient nature of the samples. Size-exclusion chromatography (SEC) coupled with SAXS (SEC-SAXS) has emerged as a valuable tool to mitigate these difficulties (David & Pérez, 2009 ▸; Pérez & Nishino, 2012 ▸; Perez & Vachette, 2017 ▸) and has been successfully integrated at most synchrotron SAXS beamlines with a focus on biological SAXS (BioSAXS) (Mathew *et al.*, 2004 ▸; David & Pérez, 2009 ▸; Watanabe & Inoko, 2009 ▸; Gunn *et al.*, 2011 ▸; Graewert *et al.*, 2015 ▸; Brennich *et al.*, 2017 ▸; Ryan *et al.*, 2018 ▸). The technique has since resulted in prominent publications (*e.g.* Berthaud *et al.*, 2012 ▸; Meisburger *et al.*, 2016 ▸; Pflüger *et al.*, 2018 ▸) and is employed by an increasing number of synchrotron users. The availability of synchrotron beamtime is, though, limited, despite great efforts from beamline administrators to adapt to user needs, and is strictly confined to the scheduled time. Samples should thus be optimized as much as possible ahead of synchrotron experiments. However, the final optimization often needs to be done during the SEC-SAXS experiment and is an iterative process. With beamtime typically allocated only a few times per year, such optimization often becomes tedious and lengthy. The workflow of BioSAXS laboratories would therefore be significantly expedited by the possibility to optimize samples at the home institution. Additionally, the high X-ray flux of synchrotrons may induce radiation damage in the samples. While attenuating the beam resolves this issue, it also leads to longer measurement times and, correspondingly, consumption of precious beamtime.

In some cases, it can be difficult to obtain complete separation of the proteins eluting from the size-exclusion column (so-called baseline separation), leaving the eluting protein solutions polydisperse. In general, separation of the individual protein peaks improves with lower flow rates (Cheng & Hollis, 1987 ▸; Ricker & Sandoval, 1996 ▸; Hong *et al.*, 2012 ▸). This, however, again leads to increased spending of beamtime. Significant efforts are therefore being made to develop data processing tools to deal with overlapping peaks. In cases where there is sufficient separation of the eluting species, it is possible to identify data regions corresponding to monodisperse samples, which may be further analyzed (Malaby *et al.*, 2015 ▸; Panjkovich & Svergun, 2018 ▸). On the other hand, in cases where there is significant overlap of the eluting species, this approach will result in the unwanted effect of discarding a major portion of the collected data (corresponding to the data from a mixture of species); it is then necessary to decompose the data before further data evaluation (Brookes *et al.*, 2013 ▸, 2016 ▸), which often poses analytical challenges (Brookes *et al.*, 2016 ▸; Herranz-Trillo *et al.*, 2017 ▸). It is thus highly desirable to optimize sample conditions and flow rates to achieve better baseline separation in order to fully exploit the potential of SEC-SAXS.

Hence, it would be of great value to the field to be able to perform SEC-SAXS on an in-house instrument which is close to the home laboratory and where experiments are not affected by radiation damage or time limitations to the same extent as on synchrotron BioSAXS beamlines. Over the past decade, advances in the development of laboratory SAXS instruments optimized for solution measurements have enabled investigation of biological samples using static (not coupled to in-line chromatography) SAXS at the home laboratory (Mortuza *et al.*, 2014 ▸; Sibillano *et al.*, 2014 ▸; Dupont *et al.*, 2015 ▸; Bruetzel *et al.*, 2016 ▸; Malmos *et al.*, 2016 ▸; Mortensen *et al.*, 2017 ▸). To our knowledge, however, there are to date no reports of SEC-SAXS having been implemented on any laboratory instruments. This is probably because of the comparatively low X-ray flux provided by classical generators, inducing the necessity for longer exposures on more concentrated samples, which is not compatible with SEC-SAXS. Although it has been suggested that laboratory-based SEC-SAXS should indeed be feasible with modern detectors (Wright *et al.*, 2013 ▸), it has recently been stated by Ryan *et al.* (2018 ▸) that ‘in-line SEC-SAXS […] is generally beyond the capability of current laboratory SAXS instruments for most proteins’.

Nevertheless, having access to a state-of-the-art laboratory BioSAXS instrument (a Xenocs BioXolver L, equipped with a powerful MetalJet X-ray source and a single-photon-counting detector), we have devised a proof-of-concept study to assess the feasibility of laboratory-based SEC-SAXS. Using an array of proteins covering a wide range of molecular weights, we demonstrate that laboratory-based SEC-SAXS yields data of sufficient statistical quality within the time used to perform a standard SEC run, while consuming no more protein than is routinely used for synchrotron SEC-SAXS measurements (typically a few milligrams). We demonstrate that laboratory-based SEC-SAXS can produce publication quality data and highlight additional advantages related to the use of a laboratory setup.

## 2. Methods   

### 2.1. Sample preparation   

All proteins were from commercial sources. Ribonuclease A (RNase A), carbonic anhydrase (CAH), ovalbumin (OVA) and conalbumin (CA) were purchased from GE Healthcare. Bovine serum albumin (BSA) and horse apoferritin (HAF) were obtained from Sigma–Aldrich. Human insulin (HI) was obtained from Novo Nordisk A/S as a zinc-free powder and formulated according to the protocol described by Nygaard *et al.* (2012 ▸), except that the initial pH lowering described by Nygaard *et al.* was avoided. Hence, after the protein had been dissolved in water, Zn(OAc)_2_, phenol, NaCl and sodium phosphate buffer were added and the concentration adjusted to reach a concentration of 600 µ*M* HI in 7 m*M* sodium phosphate (pH 7.4), 60 m*M* phenol, 200 µ*M* Zn(OAc)_2_ and 23 m*M* NaCl. The pH of the sample was then checked and gently adjusted to pH 7.4 using small amounts of HCl or NaOH. Static SAXS measurements were performed on BSA in 50 m*M* HEPES buffer at pH 7.5 prepared from lyophilized protein without additional purification. Protein concentrations were determined using UV/vis spectroscopy at 280 nm on a NanoDrop 1000 using the protein extinction coefficients in Table S1 in the supporting information (SI). In the case of the static BSA measurement, the sample concentration was additionally determined using the in-line UV/vis capabilities of the SAXS instrument.

### 2.2. Size-exclusion chromatography   

SEC was performed using an ÄKTA Purifier 100 high-performance liquid chromatography (HPLC) system from GE Healthcare coupled with a Superdex 200 Increase 10/300 GL column with a bed volume of 24 ml. In all instances, 0.5 ml of sample per measurement were loaded on the column. The flow rate was set to 0.5 ml min^−1^ and decreased to 0.1 ml min^−1^ when the protein eluted from the column, in order to ensure long enough exposure times and accordingly better counting statistics in the obtained data. The SEC runs were performed with the column kept at room temperature.

### 2.3. Small-angle X-ray scattering, laboratory   

SAXS experiments were performed on a BioXolver L, a commercial laboratory instrument from Xenocs (http://xenocs.com/en/solutions/bioxolver/), equipped with a 250 W liquid gallium alloy X-ray source (MetalJet) with wavelength λ = 1.34 Å, and a BioCUBE, a temperature-controlled flow-through cell which allows UV/vis measurements directly on the SAXS exposure volume. The flux at the sample position was of the order of 3 × 10^8^ photons s^−1^ with a beam size of approximately 1 × 1 mm. SAXS data were collected at 298 K in 30 s exposures with variable sample–detector distance, *d*, altering the probed scattering angle θ and thus the range of the scattering vector **q** [|**q**| = *q* = (4π/λ)sin(θ/2)]. Two different medium-resolution settings were used for the experiments, depending on the desired *q* range: *d* = 654 mm, *q* = (0.011–0.50) Å^−1^, suitable for most proteins, and *d* = 1507 mm, *q* = (0.0075–0.22) Å^−1^, necessary for larger macromolecules. The sample concentration during the SEC-SAXS experiment was monitored using UV absorption at 280 nm, performed directly on the SAXS exposure volume. For comparison, a static SAXS measurement was performed on a 5 mg ml^−1^ BSA sample at *d* = 654 mm, with a sample volume of 5 µl and an exposure time of 60 s.

### 2.4. Small-angle X-ray scattering, synchrotron   

Static synchrotron SAXS data were collected on 4 mg ml^−1^ BSA at the EMBL beamline P12 at Petra III in Hamburg, Germany (Blanchet *et al.*, 2015 ▸), at 293 K, covering a *q* range from 0.0023 to 0.51 Å^−1^. The exposure time was 1 s, and the sample volume of 25 µl was flowed through the beam during exposure in order to reduce radiation damage. A synchrotron SEC-SAXS experiment was performed at 283 K at the BioSAXS beamline BM29 at ESRF, Grenoble (Pernot *et al.*, 2013 ▸), using the same column as for the laboratory experiments and an 8 mg ml^−1^ BSA sample (in PBS buffer), a loading volume of 500 µl, a beam attenuation of 45% and an exposure time of 1 s per frame. In order to reduce radiation damage, 1 m*M* dithiothreitol (DTT) was used, rather than glycerol. The advantage of DTT over glycerol is that it barely affects the scattering contrast (see SI for a detailed calculation), although in some cases it might affect the structure of the protein (Jeffries *et al.*, 2015 ▸).

### 2.5. Data analysis   

The two-dimensional images were radially averaged using the software *RAW* (Nielsen *et al.*, 2009 ▸; Hopkins *et al.*, 2017 ▸). The resulting one-dimensional curves were integrated in the region between *q* = 0.05 and 0.1 Å^−1^ and the integrated intensity was plotted as a function of time. The resulting curve was used to identify the frames corresponding to the eluting protein. The numbers of frames used for the data analysis are shown in Table S1 and selected frames in Figs. S1 and S2 in the SI. Background subtraction was performed by selecting and averaging frames corresponding to the background before and after the protein elution (see Figs. S1 and S2 in the SI). No further correction of the background subtraction was necessary. Measurements for absolute scale calibration were not performed for the SEC-SAXS measurements. For each individual frame over the monomer peak, the radius of gyration, *R*_g_, and the scattering intensity in the forward direction, *I*(0), were determined by the Guinier approximation through *AUTORG* (Petoukhov *et al.*, 2007 ▸). Theoretical scattering patterns were calculated from the crystal structures to the maximum experimentally measured *q* (listed in Table S1 in the SI) using *CRYSOL* (Svergun *et al.*, 1995 ▸) with background corrections enabled. The corresponding pair-distance distribution functions, *p*(*r*), radii of gyration, 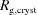, and longest extensions, 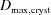 were extracted as described by Midtgaard *et al.* (2018 ▸). For the experimental data, pair-distance distribution functions, *p*(*r*), longest extensions, 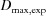, radii of gyration, 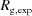, numbers of Shannon channels, *N*_s_, and so-called numbers of good parameters, *N*_p_, were determined using *BAYESapp* (Vestergaard & Hansen, 2006 ▸; Hansen, 2012 ▸). The experimental molecular weights, 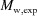, were estimated from the Porod volume using *SAXSMoW* (Fischer *et al.*, 2010 ▸). For the static BSA sample measured on the BioXolver L, the molecular weight was also determined from scattering data on absolute scale in the software *RAW* after normalization of the recorded two-dimensional images by transmitted intensity of the direct beam, measured directly on the beamstop-free detector. A pure water sample was used as a secondary standard (Orthaber *et al.*, 2000 ▸). The useful *q* range was estimated using the Shannon-channel-based approach implemented in the program *Shanum* (Konarev & Svergun, 2015 ▸). For the generation of *ab initio* models, *DAMMIF* (Franke & Svergun, 2009 ▸) was used (see Table S1 for details), run ten times, aligned and filtered. The presented models are the end results from *damfilt* (Volkov & Svergun, 2003 ▸) of the ten aligned models, visualized by *PyMol* (https://pymol.org/) with their respective known crystal structures.

## 3. Results and discussion   

### 3.1. The SEC-SAXS setup   

**Figure 1:**
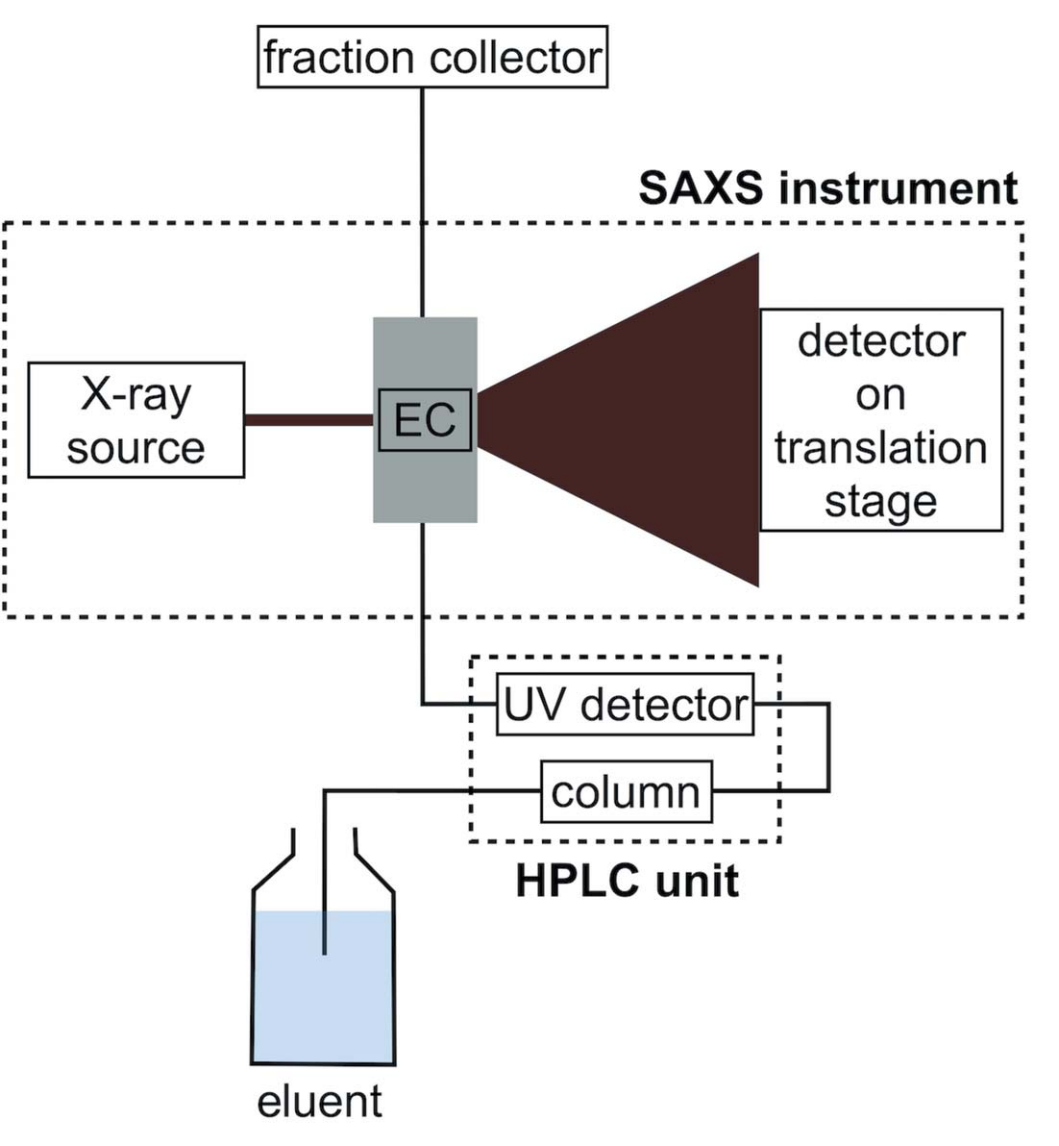
Schematic of our laboratory-based SEC-SAXS setup with the HPLC unit, composed of an ÄKTA chromatography system, a SEC column and a UV detector, the SAXS instrument (Xenocs BioXolver L) with an exposure cell (EC) for UV/vis and SAXS measurements, the X-ray source and the detector on a translation stage, and the fraction collector for sample collection after SEC-SAXS.

The instrumental setup, schematically outlined in Fig. 1 ▸, consisted of the aforementioned HPLC unit (ÄKTA Purifier 100 and SEC column) mounted on a mobile table placed in close proximity to the SAXS instrument (BioXolver L) and connected to the flow-through cell (BioCUBE) which allows UV/vis measurements directly on the SAXS exposure volume (see Fig. S3 in the SI for photographs of the actual setup). The tubing volume from the column to the point of X-ray exposure was 415 µl. A fraction collector was placed immediately after the SAXS exposure cell to collect samples for further analysis. Synchrotron setups are typically placed in a safety interlock system which can only be accessed upon completion of the experiment, after closing the shutter in front of the X-ray beam. With a laboratory-based setup, however, access to the instrument during the course of the measurements offers the option to immediately collect the individual samples for further complementary analysis as they elute from the X-ray experiment. This is particularly important for labile samples. The X-ray exposure cell is temperature controlled, and additional temperature control on buffers and samples is feasible, but not implemented in this study.

### 3.2. Sample demands and data quality   

A 60 s static SAXS measurement (without the SEC setup) on a 5 mg ml^−1^ BSA sample on the BioXolver L (Table 1 ▸) contains 3.9 good parameters, *N*_p_. For reference, an equivalent static synchrotron data set is found to have 7.9 good parameters, corresponding to a clearly more information-rich data set. This is to be expected from the larger *q* range, due to the larger detector, and five orders of magnitude higher X-ray flux available at the beamline used. Nonetheless, the result from the laboratory instrument shows that data with good enough statistical quality can be obtained with short enough exposure times to enable SEC-SAXS.

**Figure 2:**
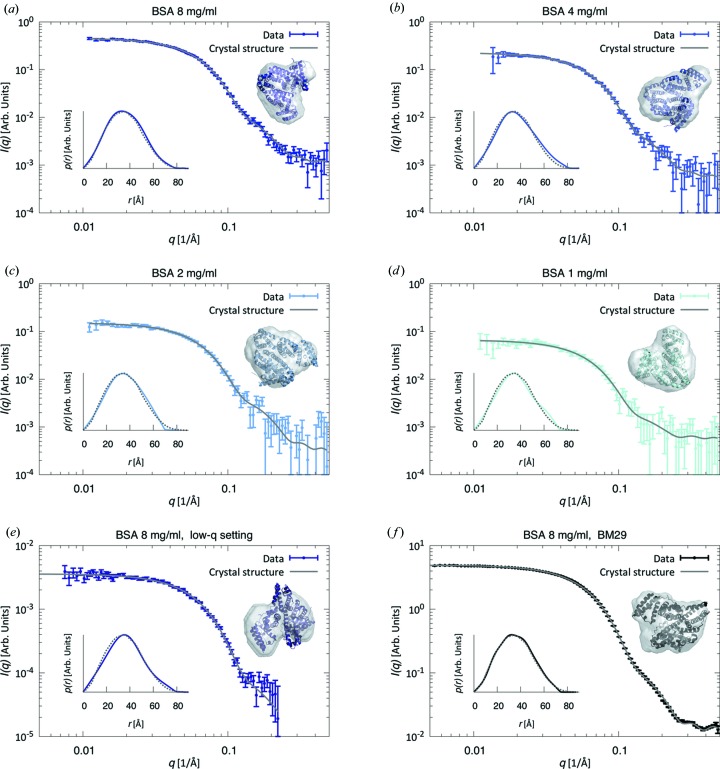
(*a*)–(*e*) BSA monomer data from SEC-SAXS (data points) at different concentrations and detector settings [(*a*)–(*d*) *d* = 654 mm, *q* = (0.011–0.50) Å^−1^; (*e*) *d* = 1507 mm, *q* = (0.0075–0.22) Å^−1^], together with the theoretical scattering curves calculated from the known crystal structure of monomeric BSA (gray lines). Insets show *ab initio* models based on the experimental data together with the crystal structure of BSA, viewed from different angles, as well as the corresponding pair-distance distribution functions, *p*(*r*) [solid lines: experimental data; dotted lines: calculated *p*(*r*)]. (*f*) SEC-SAXS BSA monomer data set from a synchrotron BioSAXS beamline (BM29, ESRF, Grenoble).

To evaluate the sensitivity of the system, a series of measurements were performed using BSA, a 66 kDa protein typically used as a calibration standard for SAXS experiments. The measurements were performed using the medium-*q* setting and covered a range of protein stock concentrations (8, 4, 2 and 1 mg ml^−1^) with loaded volumes of 0.5 ml. The radii of gyration, *R*_g_, and the forward scattering intensities, *I*(0), of the individual frames across the monomer peak of the SEC-SAXS data sets (Fig. S4 in the SI), obtained from a Guinier analysis of each frame, demonstrate the monodispersity of the samples, allowing us to average multiple exposures to improve data quality for further analysis. The results are shown in Figs. 2 ▸(*a*)–2 ▸(*d*), with the corresponding Guinier plots shown in Figs. S5A–S5D in the SI. To evaluate the data quality from the instrumental low-*q* setting, resulting in a reduced X-ray flux, the experiment was repeated in this setting for the most concentrated sample (Figs. 2*e*, S4E and S5E). For comparison, we also show a SEC-SAXS data set of 8 mg ml^−1^ BSA obtained with a loading volume of 0.5 ml at the synchrotron BioSAXS beamline BM29, ESRF, Grenoble (Figs. 2 ▸ *f*, S4F and S5F). In addition, we collected static SAXS data (*i.e.* without the SEC setup) from a sample at 5 mg ml^−1^ on our laboratory setup and 4 mg ml^−1^ at the beamline P12 (data shown in Fig. S6 in the SI and the corresponding Guinier plots in Figs. S5G–S5H). All data correspond well to the theoretically calculated curves based on the crystal structure of monomeric BSA (Fig. 2 ▸), evaluating parameters both in reciprocal [*I*(*q*)] and in direct space [*p*(*r*)]. The experimentally determined molecular weights 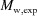 from *SAXSMoW* yield values within less than 10% of the theoretical value of 66 kDa (Table 1 ▸).

In an effort to quantify the data quality, we determined the number of Shannon channels, *N*_s_, and good parameters, *N*_p_, in the SEC-SAXS data and reference static SAXS measurements, as well as the usable *q* range. These values are reported in Table 1 ▸, together with the experimentally determined structural parameters. As expected, the number of Shannon channels is roughly constant for data collected with the same detector setting (the variation between the values is caused by changes of the experimental value of 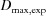). The number of good parameters, however, is a better measure of the information content. Here, we observe a clear correlation between the noise level of the data and the number of good parameters available (Vestergaard & Hansen, 2006 ▸; Hansen, 2012 ▸; Pedersen *et al.*, 2014 ▸). In the data set from 8 mg ml^−1^ BSA, 4.3 good parameters are found available in the data, gradually decreasing to 2.4 for the 1 mg ml^−1^ BSA data, clearly demonstrating the lower data quality associated with the lower protein concentration. Estimating the data quality by the largest meaningful *q* value from *Shanum*, it is also evident from Table 1 ▸ that the high-*q* region of the data is useful for all measurements, except for the lowest concentration. The SEC-SAXS data set from a synchrotron beamline from 8 mg ml^−1^ BSA (Fig. 2 ▸ *f*) contains 8.6 good parameters and a maximum useful *q* of 0.48 Å^−1^, again associated with the higher X-ray flux of the synchrotron.

Overall, the analysis presented here demonstrates that our setup produces data of good statistical quality over a range of protein concentrations and sample–detector distances. Ultimately, the minimum loaded amount of material guaranteeing sufficient data quality depends on the protein size, as well as the monodispersity of the sample, since higher polydispersity implies a lower total amount of protein in the main elution peak.

### 3.3. Molecular weight range   

**Figure 3:**
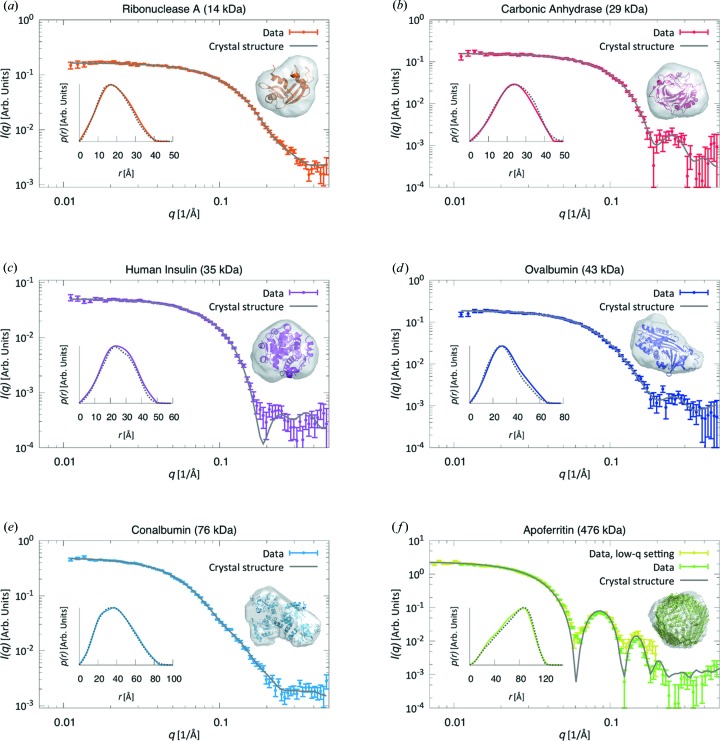
(*a*)–(*e*) SEC-SAXS data sets (data points) of various proteins, together with the *ab initio* models obtained from the experimental data and the known crystal structures for comparison. Also shown are the theoretical scattering curves (gray lines) calculated from the latter. Insets: pair-distance distribution functions *p*(*r*) from the experimental data (solid lines) and from the crystal structures (dotted lines). (*f*) The low-*q* data were scaled to overlap with the data from the high-*q* setting. The *CRYSOL* fit, *p*(*r*) and *ab initio* model were calculated on the basis of merged data from both settings.

Having established the sensitivity of our setup, we demonstrate the general applicability of laboratory SEC-SAXS by presenting data from six additional proteins (Figs. 3 ▸, S7 and S8 and Table 2 ▸), spanning a range of molecular weights from 14 to 476 kDa, and applying two different sample–detector distances for the largest protein (apoferritin) (see Table S1 in the SI for details).

The scattering curves of all proteins are consistent with those calculated from their respective crystal structures and the data contain between 2.7 and 6.9 good parameters, *N*_p_, depending on the size of the protein (larger proteins have larger *N*_p_) and the concentration of the sample. All measured radii of gyration (listed in Table 2 ▸) are in agreement with values obtained from the crystal structures, and the molecular weights estimated from the experimental data using *SAXSMoW* are in good agreement with the known molecular weights (Tables 1 ▸ and 2 ▸). The size of the X-ray beam leads to a small instrumental smearing on laboratory sources. Such resolution effects, present in the experimental data, were not included in the *CRYSOL* fits presented here, leading to the sharper features of the theoretical scattering curves in Figs. 3 ▸(*c*) and 3 ▸(*f*) compared to the experimental data.

Fig. 3 ▸(*f*) shows good agreement between data obtained with different sample–detector distances and demonstrates the potential to cover a broader *q* range by merging data sets from different settings.

### 3.4. Direct UV/vis measurements on the SAXS exposure cell   

**Figure 4:**
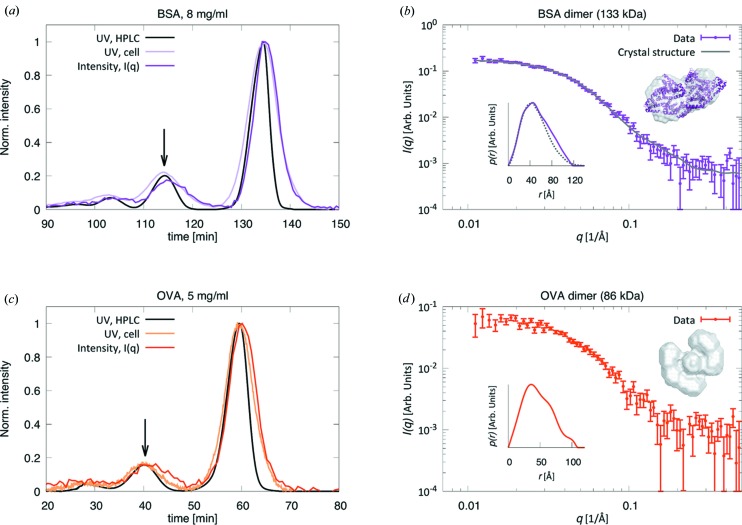
Data for (*a*), (*b*) BSA and (*c*), (*d*) OVA. (*a*), (*c*) UV traces (at 280 nm) from the HPLC unit (black lines) and on the SAXS exposure cell (light purple and light orange), together with the integrated SAXS intensity *I*(*q*) over time (dark purple and dark orange). Arrows indicate the dimer elution peaks. (*b*), (*d*) SEC-SAXS data sets (data points) corresponding to the protein dimers, marked by the arrows in panels (*a*) and (*c*), and theoretical scattering curve calculated from the known crystallographic dimer structure of BSA (gray line), together with the corresponding pair-distance distribution functions, *p*(*r*), from the experimental data (solid lines) and from the crystal structure (dotted line). *Ab initio* models obtained from the experimental data and the crystal structure (for BSA) are shown as insets.

Using low flow rates, we obtain baseline separation of monomer and dimer elution peaks in all cases (see Fig. 4 ▸ for two examples, BSA and OVA), except for HAF, the largest of the proteins investigated here. The concentrations of the protein dimers are significantly lower than those of the corresponding monomers, evident from the UV traces shown in Figs. 4 ▸(*a*) and 4 ▸(*c*).

Broadening of the eluting protein peak always happens in a SEC-SAXS experiment owing to the large difference in diameter between standard HPLC tubing and the SAXS capillary and to Taylor dispersion in the tubing (Taylor, 1953 ▸). Correct estimation of this broadening is crucial for accurate concentration determination of the sample, necessary for direct molecular weight determination from the scattering data (Mylonas & Svergun, 2007 ▸). The ability of our setup to perform UV/vis measurements directly on the SAXS exposure cell, in conjunction with SAXS data acquisition, enables accurate determination of the chromatogram, and thus the protein concentration, at the point of exposure (Figs. 4 ▸ *a* and 4 ▸ *c*). In fact, the UV trace from the SAXS exposure cell presents a wider peak than that from the HPLC unit and overlaps with the integrated SAXS intensity. Establishment of the exact SEC-SAXS chromatogram allows a full correspondence between the fractionated samples after SAXS exposure and the corresponding measured SAXS data. Further characterization by additional biophysical or biochemical techniques can thus be linked directly to potential structural differences in the eluting proteins.

As measurements for absolute scale calibration were not performed during this study, we instead demonstrate the accuracy of the direct molecular weight determination from scattering data on absolute scale by means of a static BSA measurement performed on the same instrument (Fig. S6 in the SI). The in-line UV/vis and the NanoDrop spectrophotometer indeed yield the same value for the protein concentration (4.5 mg ml^−1^), and the experimentally determined molecular weight from data on absolute scale (64 kDa) differs by less than 5% from the known molecular weight of BSA (66 kDa).

We demonstrate that the relatively high X-ray flux and low background-to-noise level of our laboratory setup yield data of sufficient statistical quality to even enable structural investigation of minor solution components, as illustrated by the BSA and OVA dimers (Table 3 ▸). While the peak broadening associated with increased flow rates might not be an impediment if SEC is merely employed to separate monomeric protein from higher oligomer species, more complex samples may require optimized peak resolution and thus low flow rates. Here, laboratory-based SEC-SAXS optimally complements synchrotron setups.

## 4. General discussion   

Synchrotron SEC-SAXS is in many aspects superior to laboratory-based experiments, given the high flux, allowing for a higher signal-to-noise ratio, the more focused beam and hence minimal smearing effects, and the wider *q* range through larger detectors. However, laboratory-based SEC-SAXS has some intrinsic advantages that should be mentioned here. Synchrotron SEC-SAXS requires transportation of samples to the beamline, with inherent challenges related to the sensitivity of biological samples to temperature differences, mechanical stress and time. Particularly short-lived perishable samples might even necessitate preparation on site, thus requiring dedicated facilities at the synchrotron (Boivin *et al.*, 2016 ▸). In conjunction with the time pressure associated with synchrotron experiments, necessary extensive sample handling on site increases the risk of compromising the experiments. In the current study, we have used standard proteins for a proof-of-concept study, but it follows that vulnerable samples would greatly benefit from the ability to perform SEC-SAXS experiments on a laboratory-based instrument, immediately following careful and optimized preparation. In addition, easy access and available space around laboratory instruments, compared to synchrotron setups, in general allow for more complicated setups on the sample side and give additional flexibility when optimizing experiments. In particular, it is possible to collect the fractionated samples directly after the SAXS measurements and subject them to further biochemical or biophysical analysis. Given the accurate UV/vis assessment of the elution profile directly on the SAXS exposure cell, the results can be directly correlated with potential structural differences in each fraction, detected *via* the SAXS analysis.

Even for samples where the improved conditions on synchrotron beamlines are needed, laboratory-based SEC-SAXS evidently can serve as a valuable tool for initial investigations and optimization of samples prior to synchrotron SEC-SAXS.

Finally, small-angle neutron scattering (SANS) is gradually gaining in popularity in the biostructural research field (Chaudhuri, 2015 ▸; Gabel, 2015 ▸) and significant development is taking place, now also enabling implementation of in-line SEC (SEC-SANS) (Jordan *et al.*, 2016 ▸). SANS is complementary to SAXS, yielding similar information but with the added benefit of being able to distinguish between different components of a solution through contrast variation (Skar-Gislinge *et al.*, 2010 ▸; Hennig *et al.*, 2013 ▸; Kynde *et al.*, 2014 ▸). It is therefore frequently used in conjunction with SAXS at large-scale facilities. Bar a few exceptions, this typically requires multiple experiments at different facilities. This not only renders such experiments logistically challenging, but may also lead to data acquired on different samples, as many biological samples need to be freshly prepared immediately prior to the experiment. SANS beamlines starting to offer SEC-SANS to investigate complex and/or unstable biological samples would thus greatly benefit from having a laboratory-based SAXS instrument with an integrated SEC-SAXS setup directly on site. This would enable users to perform both experiments in parallel, on the same sample, thus avoiding ambiguity related to sample variations.

## 5. Conclusion   

We demonstrate that it is possible to perform SEC-SAXS on a laboratory-based instrument, provided it is optimized for high X-ray flux and signal-to-noise ratio. Recovery of the sample for further investigation using complementary techniques is possible immediately following SEC-SAXS, and thus users benefit from the presence of additional biophysics and biochemistry instruments at the home institution. UV/vis measurements on the SAXS exposure cell furthermore allow in-line concentration determination during the SEC-SAXS experiment and accurate correlation between SAXS data and the fractionated samples, obtained *via* correct estimation of the peak broadening. This enhances the opportunity for assignment of structure–function relationships. The laboratory-based setup presented here can be used not only to optimally prepare for synchrotron SEC-SAXS by optimizing samples at the home institution but also as an alternative to synchrotron experiments for a vast range of samples, as the obtained data are demonstrated here to be of useful statistical quality, leaving the precious synchrotron beamtime for samples requiring the higher flux or broader *q* range. Available measurement time not being as much a concern on a laboratory-based instrument as it is on synchrotrons, the former can also serve as a tool to investigate complex samples with multiple components which require slow flow rates for good baseline resolution.

## Supplementary Material

Table with details of experiments, analysis and results, contrast calculation, additional figures (individual frames, frame selection, frame-by-frame analysis, Guinier plots, static data), and pictures of the experimental setup. DOI: 10.1107/S1600576718014462/vg5091sup1.pdf

## Figures and Tables

**Table 1:** Data quality and structural parameters Data quality and structural parameters from the experimental SEC-SAXS data of BSA. Also shown are the values obtained from static measurements on our laboratory instrument and a synchrotron BioSAXS beamline (P12, EMBL-Hamburg), as well as from a SEC-SAXS experiment on the beamline BM29, ESRF, Grenoble, for comparison. *N*_s_: number of Shannon channels; *N*_p_: number of good parameters; 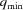 and 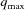: smallest and largest measured *q*; 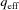: largest effectively useful *q* (from *Shanum*); 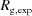: experimentally determined radius of gyration (from *BAYESapp*); 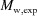: experimentally determined molecular weight (from *SAXSMoW*); 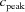: protein concentration at the maximum of the elution peak. Numbers in parentheses are uncertainties on the least significant digit.

Sample	*N*_*s*_	*N*_p_	*q*_min_ (Å^−1^)	*q*_max_ (Å^−1^)	*q*_eff_ (Å^−1^)	*R*_g,exp_ (Å)	*M*_w,exp_ (kDa)	*c*_peak_ (mg ml^−1^)
7.6 mg ml^−1^	5.2	4.3 (2)	0.011	0.50	0.50	27.5 (1)	65	3.8
3.7 mg ml^−1^	5.3	3.4 (3)	0.011	0.50	0.46	28.3 (1)	67	1.8
2.0 mg ml^−1^	4.5	2.7 (3)	0.011	0.50	0.46	27.1 (1)	64	0.9
1.0 mg ml^−1^	4.8	2.4 (3)	0.011	0.50	0.40	27.5 (2)	61	0.4
7.6 mg ml^−1^, low *q*	4.8	2.4 (5)	0.0075	0.22	0.19	27.8 (2)	61	3.8
4.6 mg ml^−1^, static	6.0	3.9 (2)	0.011	0.45	0.33	28.4 (1)	66	n/a
4.3 mg ml^−1^, static, P12	9.4	7.9 (2)	0.0023	0.51	0.50	28.0 (1)	66	n/a
8.1 mg ml^−1^, BM29	11.1	8.6 (4)	0.0054	0.48	0.48	27.1 (1)	63	n/a

**Table 2:** Structural and other parameters of various proteins Structural parameters of various proteins calculated on the basis of the scattering profiles from the SEC-SAXS experiments [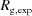 (from *BAYESapp*) and 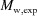 (from *SAXSMoW*)] and from the known crystal structures (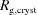), as well as the known molecular weight of the proteins (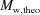). Also shown are the number of good parameters, *N*_p_, and the largest effectively useful *q* (from *Shanum*), 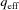, of the experimental scattering data, together with the smallest and largest measured *q*, 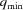 and 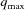, and the concentration at the maximum of the elution peak, 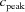, determined using the UV/vis detection on the SAXS exposure cell. Note that for HI no peak concentration is available because of the presence of phenol, which strongly absorbs at 280 nm, in the running buffer. Numbers in parentheses are uncertainties on the least significant digit.

Protein	*R*_g,exp_ (Å)	*R*_g,cryst_ (Å)	*M*_w,exp_ (kDa)	*M*_w,theo_ (kDa)	*N*_*p*_	*q*_min_ (Å^−1^)	*q*_max_ (Å^−1^)	*q*_eff_ (Å^−1^)	*c*_peak_ (mg ml^−1^)
RNase A	14.7 (1)	14.4	10	14	3.7 (6)	0.011	0.50	0.46	4.2
CAH	17.9 (1)	18.3	31	29	2.7 (1)	0.011	0.50	0.46	2.5
HI	19.1 (1)	18.8	29	35	3.7 (1)	0.011	0.50	0.49	n/a
OVA	23.7 (1)	22.8	40	43	3.5 (5)	0.011	0.50	0.43	2.0
CA	30.4 (1)	30.3	76	76	5.0 (5)	0.011	0.50	0.50	2.6
HAF	52.0 (1)	53.0	434	476	6.9 (2)	0.011	0.50	0.49	3.7
HAF, low *q*	52.3 (1)	53.0	485	476	4.4 (5)	0.0075	0.22	0.19	3.7
HAF, merged	52.2 (1)	53.0	437	476	6.2 (2)	0.0075	0.50	0.47	3.7

**Table 3:** Structural and data validation parameters of BSA and OVA dimers 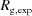
: radius of gyration (from *BAYESapp*); *N*_p_: number of good parameters; 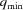 and 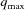: smallest and largest measured *q*; 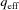: largest effectively useful *q* (from *Shanum*). 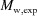: molecular weight from *SAXSMoW*. For the OVA dimer, the software was not able to calculate a molecular weight from the experimental data. Numbers in parentheses are uncertainties on the least significant digit.

Protein	*R*_g,exp_ (Å)	*M*_w,exp_ (kDa)	*N*_p_	*q*_min_ (Å^−1^)	*q*_max_ (Å^−1^)	*q*_eff_ (Å^−1^)
BSA dimer	40.8 (2)	134	3.5 (4)	0.011	0.50	0.43
OVA dimer	36.4 (2)	n/a	2.8 (3)	0.011	0.50	0.43
