# Supplementary material for: Size-exclusion chromatography small-angle X-ray scattering of water soluble proteins on a laboratory instrument
Source: J Appl Crystallogr. 2018 Nov 9;51(Pt 6):1623–32. doi: 10.1107/S1600576718014462 (PMC6276278; doi:10.1107/S1600576718014462)
Supplement: Supplementary file 1 [file j-51-01623-sup1.pdf]

Size-exclusion chromatography small-angle X-ray scattering (SEC-SAXS) of water soluble proteins on a laboratory instrument
Supporting Information

Saskia Bucciarelli, Søren Roi Midtgaard, Martin Nors Pedersen, Søren Skou, Lise Arleth and Bente Vestergaard

September 29, 2018

Table S1: Details of proteins, SAXS experiments and analysis.

protein [a]	BSA								BSA dimer	RNase A	CAH	HI	OVA	OVA dimer	CA	HAF	
**Samples**																	
PDB ID	4f5s [1]								4f5s	9rat [2]	1v9e [3]	1ev3 [4]	1ova [5]		1aiv [6]	1ies [7]	
Source [b]	SA								SA	GE	GE	NN [c]	GE		GE	SA	
M_w [kDa] [d]	66	66	66	66	66	66	66	66	133	14	29	35	43	86	76	476	476
ε_{280nm} [$M^{-1}cm^{-1}$] [e]	43824								n/a	9440	50420	5850	31775	n/a	88165	348000	
Buffer [f]	1	1	1	1	1	2	2	3	1	1	1	4	1	1	1	1	1
c [mg/ml] [g]	7.6	3.7	2.0	1.0	7.6	4.6	4.3	8.1	7.6	7.5	5.4	3.5	4.9	4.9	5.1	11.1	11.1
Volume [μl]	500	500	500	500	500	5	25	500	500	500	500	500	500	500	500	500	500
Amount of protein [mg]	3.8	1.9	1.0	0.5	3.8	0.023	0.11	4.1	3.8	3.8	2.7	1.8	2.5	2.5	2.6	5.6	5.6
**Data collection**																	
Instrument [h]	BX	BX	BX	BX	BX	BX	P12	BM29	BX	BX	BX	BX	BX	BX	BX	BX	BX
Detector [i]	P300K	P300K	P300K	P300K	P300K	P300K	P2M	P1M	P300K	P300K	P300K	P300K	P300K	P300K	P300K	P300K	P300K
Wavelength [Å]	1.34	1.34	1.34	1.34	1.34	1.34	1.24	0.99	1.34	1.34	1.34	1.34	1.34	1.34	1.34	1.34	1.34
Flux [ph/s] [j]	∼2.5e8	∼6e7*	∼2.5e8	∼2.5e8	∼4.2e7	∼2.5e8	∼1e13	∼4e11	∼2.5e8	∼2.5e8	∼2.5e8	∼2.5e8	∼2.5e8	∼2.5e8	∼2.5e8	∼2.5e8	∼4.2e7
d [mm] [k]	654	654	654	654	1507	654	3000	2867	654	654	654	654	654	654	654	654	1507
q_{min} [$Å^{-1}$] [l]	0.011	0.011	0.011	0.011	0.0075	0.011	0.0023	0.0054	0.011	0.011	0.011	0.011	0.011	0.011	0.011	0.011	0.0075
q_{max} [$Å^{-1}$] [m]	0.50	0.50	0.50	0.50	0.22	0.45	0.51	0.48	0.50	0.50	0.50	0.50	0.50	0.50	0.50	0.50	0.22
Exposure time [s]	30	30	30	30	30	60	1	1	30	30	30	30	30	30	30	30	30
Temperature [^{o}C]	25	25	25	25	25	25	20	10	25	25	25	25	25	25	25	25	25
Collection mode	SEC	SEC	SEC	SEC	SEC	static	static	SEC	SEC	SEC	SEC	SEC	SEC	SEC	SEC	SEC	SEC
Column size & media	10×300 S200 Inc	10×300 S200 Inc	10×300 S200 Inc	10×300 S200 Inc	10×300 S200 Inc	n/a	n/a	10×300 S200 Inc	10×300 S200 Inc	10×300 S200 Inc	10×300 S200 Inc	10×300 S200 Inc	10×300 S200 Inc	10×300 S200 Inc	10×300 S200 Inc	10×300 S200 Inc	10×300 S200 Inc
Flow rate [ml/min]	0.1	0.1	0.1	0.1	0.1	n/a	n/a	0.7	0.1	0.1	0.1	0.1	0.1	0.1	0.1	0.1	0.1
**Data analysis**																	
# of frames (protein)	12	10	10	10	13	n/a	n/a	40	9	11	10	13	11	10	10	11	11
# of frames (background)	28 + 12	25 + 20	19 + 5	19 + 10	15 + 20	n/a	n/a	200 + 100	15 + 20	40 + 20	20 + 10	50 + 6	20 + 20	20 + 20	20 + 20	20 + 10	19 + 10
Data reduction	RAW [8, 9], Python																
Estimation of q_{eff}	Shanum (ATSAS 2.8) [10]																
Guinier analysis	AUTORG [11] (individual frames), RAW (averaged data)																
IFT	BAYESapp [12, 13]																
Molecular weight determination	SAXSMoW [14]																
Ab initio modeling	DAMMIF (slow mode), damfilt (ATSAS 2.8) [15, 16]																
symmetry/anisometry	P1/n.a.	P1/n.a.	P1/n.a.	P1/n.a.	P1/n.a.	P1/n.a.	P1/n.a.	P1/n.a.	P2/prolate	P1/n.a.	P1/n.a.	P3/n.a.	P1/n.a.	P1/oblate	P1/n.a.	P1/n.a.	P1/n.a.
Computation of crystal structure intensities	CRYSOL (ATSAS 2.8) [17] with background correction enabled																
Analysis of crystal structure intensities	in-house routine [18]																
3D representation	PyMol																
**Results**																	
Peak c [mg/ml] [n]	3.8	1.8	0.9	0.4	3.8	n/a	n/a	n/a	n/a	4.2	2.5	n/a	2.0	n/a	2.6	3.7	3.7
Shanum q_{eff} [$Å^{-1}$]	0.50	0.46	0.46	0.40	0.19	0.33	0.50	0.48	0.50	0.46	0.46	0.49	0.43	0.43	0.50	0.49	0.19
CRYSOL χ^2	1.08	0.78	0.82	0.80	1.02	1.03	13.34	23.93	1.19	1.17	0.72	1.21	0.75	n/a	1.20	6.50	1.61
Guinier $I(0)$ ($\Delta I(0)$) [Arb. Units]	4.6e-1 (3.2e-3)	6.8e-2 (1.1e-3)	1.5e-1 (3.3e-3)	6.9e-2 (2.8e-3)	3.6e-3 (5.1e-5)	4.6e-2 (4.5e-4)	4.5e-2 (6.1e-3)	4.9 (1.8e-3)	1.9e-1 (5.0e-3)	1.7e-1 (1.1e-3)	1.6e-1 (1.6e-3)	5.3e-2 (4.9e-4)	1.9e-1 (2.0e-3)	7.0e-2 (4.0e-3)	4.9e-1 (3.9e-3)	2.3e0 (1.9e-2)	1.7e-2 (2.4e-4)
Guinier R_g (ΔR_g)[Å] [o]	28.3 (0.3)	28.1 (0.6)	27.7 (1.0)	29.0 (1.7)	27.9 (0.6)	28.4 (0.4)	27.7 (0.1)	27.0 (0.1)	41.1 (1.4)	14.9 (0.2)	18.6 (0.3)	20.0 (0.3)	23.5 (0.4)	36.7 (2.9)	30.1 (0.4)	51.6 (0.6)	53.2 (1.1)
BAYESapp R_g (ΔR_g) [Å]	27.5 (0.1)	28.3 (0.1)	27.1 (0.1)	27.5 (0.2)	27.8 (0.2)	28.4 (0.1)	28.0 (0.1)	27.1 (0.1)	40.8 (0.2)	14.7 (0.1)	17.9 (0.1)	19.1 (0.1)	23.7 (0.1)	36.4 (0.2)	30.4 (0.1)	52.0 (0.1)	52.3 (0.1)
Crystal R_g [Å]	27.1	27.1	27.1	27.1	27.1	27.1	27.1	27.1	38.7	14.4	18.3	18.8	22.8	n/a	30.3	53.0	53.0
BAYESapp D_{max} (ΔD_{max}) [Å] [p]	79.6 (12.2)	81.0 (9.1)	69.4 (4.7)	73.0 (7.4)	78.9 (7.9)	78.9 (4.5)	79.1 (0.5)	72.6 (0.3)	115.5 (3.2)	41.1 (6.4)	44.2 (2.6)	49.2 (1.4)	65.2 (1.3)	106.1 (0.6)	84.0 (1.2)	119.6 (0.5)	120.4 (1.7)
Crystal D_{max} [Å]	92.5	92.5	92.5	92.5	92.5	92.5	92.5	92.5	147.5	46.8	58.0	54.9	74.2	n/a	106.1	150.3	150.3
BAYESapp N_p (ΔN_p) [q]	4.3 (0.2)	3.4 (0.3)	2.7 (0.3)	2.4 (0.3)	2.4 (0.5)	3.9 (0.2)	7.9 (0.2)	8.6 (0.4)	3.2 (0.6)	3.7 (0.6)	2.7 (0.1)	3.7 (0.1)	3.5 (0.5)	2.8 (0.3)	5.0 (0.5)	6.9 (0.2)	4.4 (0.5)
BAYESapp N_s [r]	5.2	5.3	4.5	4.8	4.8	6.0	9.4	11.1	10.6	6.5	3.4	6.1	8.0	6.2	10.4	7.2	7.4
SAXSMoW M_w [kDa]	65	67	64	61	61	66	66	63	134	10	31	29	40	n/a	76	434	485

[a] Proteins: BSA: bovine serum albumin, RNase A: ribonuclease A, CAH: carbonic anhydrase, HI: human insulin, OVA: ovalbumin, CA: conalbumin, HAF: horse apoferritin

[b] GE: GE Healthcare, SA: Sigma-Aldrich, NN: Novo Nordisk A/S

[c] Formulation:
Dissolve the protein in water. Add $Zn(OAc)_2$, phenol, NaCl and sodium phosphate buffer. Adjust concentration to 600 μM HI in 7 mM sodium phosphate (pH 7.4), 60 mM phenol, 200 μM $Zn(OAc)_2$ and 23 mM NaCl. Check pH of the sample and gently adjust to pH 7.4 using small amounts of HCl or NaOH [19].

[d] M_w: molecular weight

[e] ε_{280nm}: extinction coefficient at 280 nm

[f] Buffers: 1 - PBS, pH 7.4
2 - 50 mM HEPES, pH 7.5
3 - PBS, 1 mM DTT, pH 7.4
4 - 7 mM sodium phosphate, 60 mM phenol, 200 μM $Zn(OAc)_2$, 23 mM NaCl, pH 7.4

[g] c: concentration

[h] BX: Xenocs BioXolver L (commercial laboratory instrument), P12: BioSAXS beamline P12, EMBL-Hamburg (synchrotron), BM29: BioSAXS beamline ESRF, Grenoble (synchrotron)

[i] P300K: windowless DECTRIS Pilatus 300K without beamstop, P2M: DECTRIS Pilatus 2M, P1M: DECTRIS Pilatus 1M

[j] flux at the sample position

[k] d: sample-detector distance

[l] q_{min}: smallest measured q

[m] q_{max}: largest measured q

[n] concentration at the maximum of the elution peak

[o] R_g: radius of gyration

[p] D_{max}: longest extension of the protein

[q] N_p: number of good parameters

[r] N_s: number of Shannon channels

* *NB*: for the measurement on the 4 mg/ml BSA sample, the intensity of the direct beam was lower than for the other measurements.

Contrast calculation

The scattering of a protein sample in the forward direction $I(0)$ is proportional to $(\Delta\rho)^2$, where $\Delta\rho = \rho_{\text{protein}} - \rho_{\text{buffer}}$ is the scattering contrast and ρ_{protein} and ρ_{buffer} are the scattering length densities of the protein and the buffer, respectively. They are given by

$$\rho_{\text{protein}} = \rho_{\text{e,protein}} \cdot r_0 = \rho_{\text{M,protein}} \cdot r_0/\bar{v} \qquad \text{and} \qquad \rho_{\text{buffer}} = \rho_{\text{e,buffer}} \cdot r_0 \tag{1}$$

where $r_0 = 2.82\text{e-}13$ cm is the classical electron radius, $\rho_{\text{M,protein}} = 3.22\text{e}23$ e/g [20] is the protein electron density per mass, $\bar{v} = 0.74\ \text{cm}^3/\text{g}$ is the voluminosity of the protein and $\rho_{\text{e,protein}} = 4.34\text{e}23\ \text{e/cm}^3$ and $\rho_{\text{e,buffer}}$ are the protein and buffer electron densities, respectively. Using the electron densities for PBS buffer, $\rho_{\text{e,PBS}} = 3.37\text{e}23\ \text{e/cm}^3$, and glycerol, $\rho_{\text{e,gly}} = 4.12\text{e}23\ \text{e/cm}^3$, the relative scattering contrast and forward scattering intensity in PBS with glycerol and DTT, respectively, are summarized in the following tables.

$c(\text{gly})$ [%v/v]	$\rho_{\text{e,PBS,gly}}$ [e/cm^3]	$\Delta\rho_{\text{PBS,gly}}$ [e/cm^2]	$\frac{\Delta\rho_{\text{PBS,gly}}}{\Delta\rho_{\text{PBS}}}$ [%]	$\frac{I(0)_{\text{PBS,gly}}}{I(0)_{\text{PBS}}} = \frac{(\Delta\rho_{\text{PBS,gly}})^2}{(\Delta\rho_{\text{PBS}})^2}$ [%]
1	3.38e23	2.70e10	99.2	98.5
2	3.39e23	2.68e10	98.4	96.9
3	3.39e23	2.66e10	97.7	95.4
5	3.41e23	2.62e10	96.1	92.4
7	3.42e23	2.57e10	94.6	89.4
10	3.45e23	2.51e10	92.2	85.1

$c(\text{DTT})$ [mM]	$\rho_{\text{e,PBS,DTT}}$ [e/cm^3]	$\Delta\rho_{\text{PBS,DTT}}$ [e/cm^2]	$\frac{\Delta\rho_{\text{PBS,DTT}}}{\Delta\rho_{\text{PBS}}}$ [%]	$\frac{I(0)_{\text{PBS,DTT}}}{I(0)_{\text{PBS}}} = \frac{(\Delta\rho_{\text{PBS,DTT}})^2}{(\Delta\rho_{\text{PBS}})^2}$ [%]
1	3.37e23	2.72e10	99.9	99.9
2	3.37e23	2.72e10	99.9	99.8
3	3.37e23	2.72e10	99.8	99.7
5	3.37e23	2.72e10	99.7	99.5
7	3.37e23	2.71e10	99.6	99.3
10	3.38e23	2.71e10	99.5	99.0

$\Delta\rho_{\text{PBS}}$, $\Delta\rho_{\text{PBS,gly}}$ and $\Delta\rho_{\text{PBS,DTT}}$ are the scattering contrasts of a protein in pure PBS buffer, in PBS buffer with glycerol and in PBS buffer with DTT, respectively, and $I(0)_{\text{PBS}}$, $I(0)_{\text{PBS,gly}}$ and $I(0)_{\text{PBS,DTT}}$ are the corresponding forward scattering intensities.

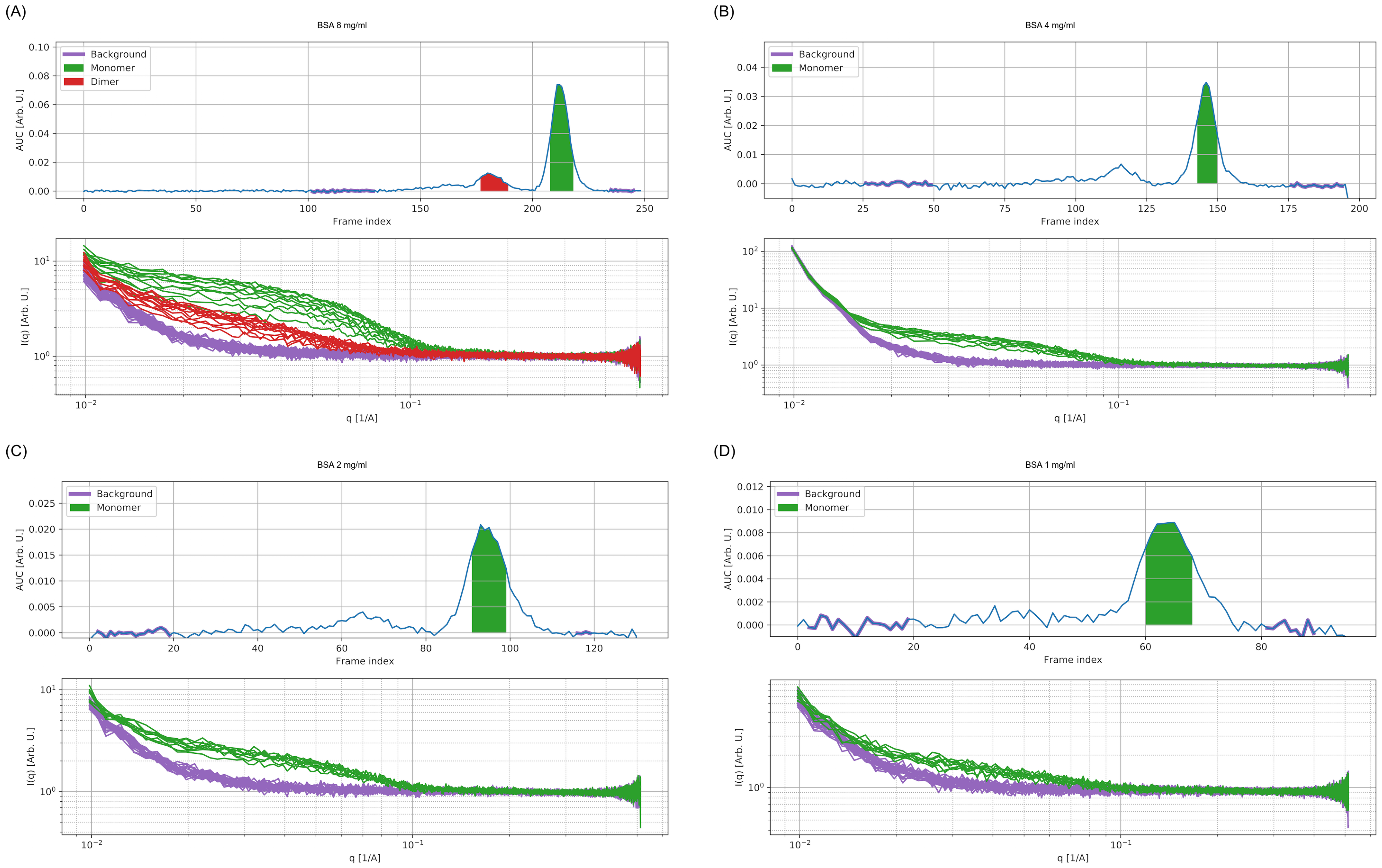

(A)
BSA 8 mg/ml
Background
Monomer
Dimer
AUC [Arb. U.]
Frame index
I(q) [Arb. U.]
q [1/A]
(B)
BSA 4 mg/ml
Background
Monomer
AUC [Arb. U.]
Frame index
I(q) [Arb. U.]
q [1/A]
(C)
BSA 2 mg/ml
Background
Monomer
AUC [Arb. U.]
Frame index
I(q) [Arb. U.]
q [1/A]
(D)
BSA 1 mg/ml
Background
Monomer
AUC [Arb. U.]
Frame index
I(q) [Arb. U.]
q [1/A]

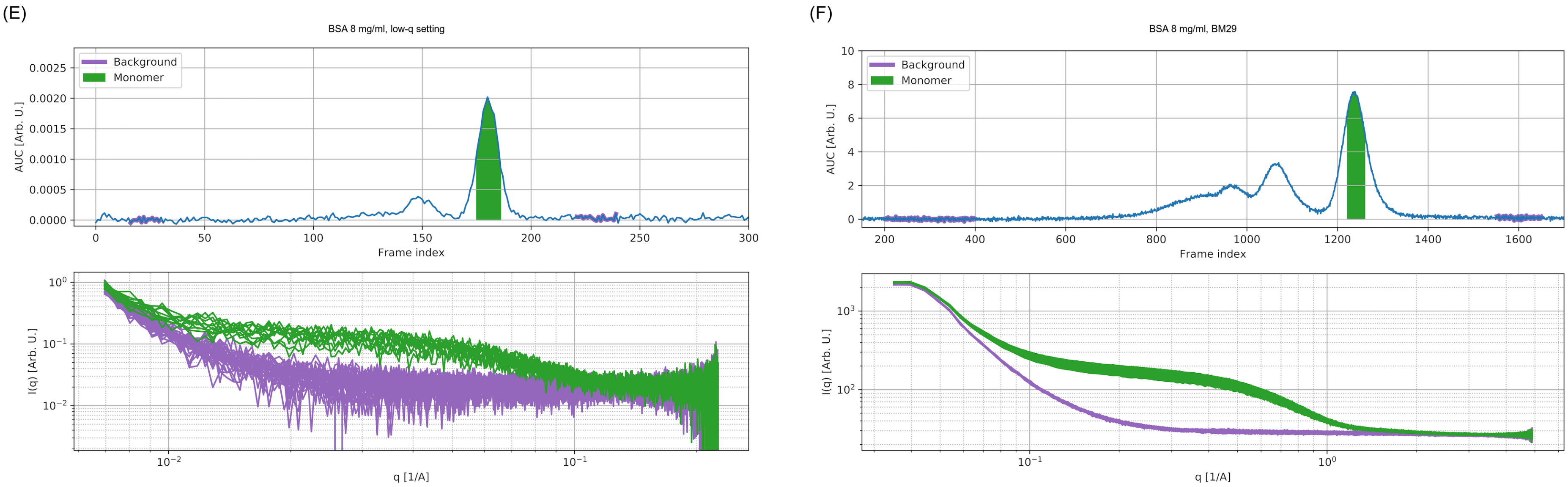

Figure S1: Selection of sample and buffer regions for data analysis of SEC-SAXS data from BSA samples with stock concentrations of 8, 4, 2 and 1 mg/ml. *Top panels:* Integrated intensity as a function of frame index (i.e. time), *bottom panels:* Individual 30s frames. A-E: Xenocs BioXolver L, F: synchrotron BioSAXS beamline BM29, ESRF-Grenoble.

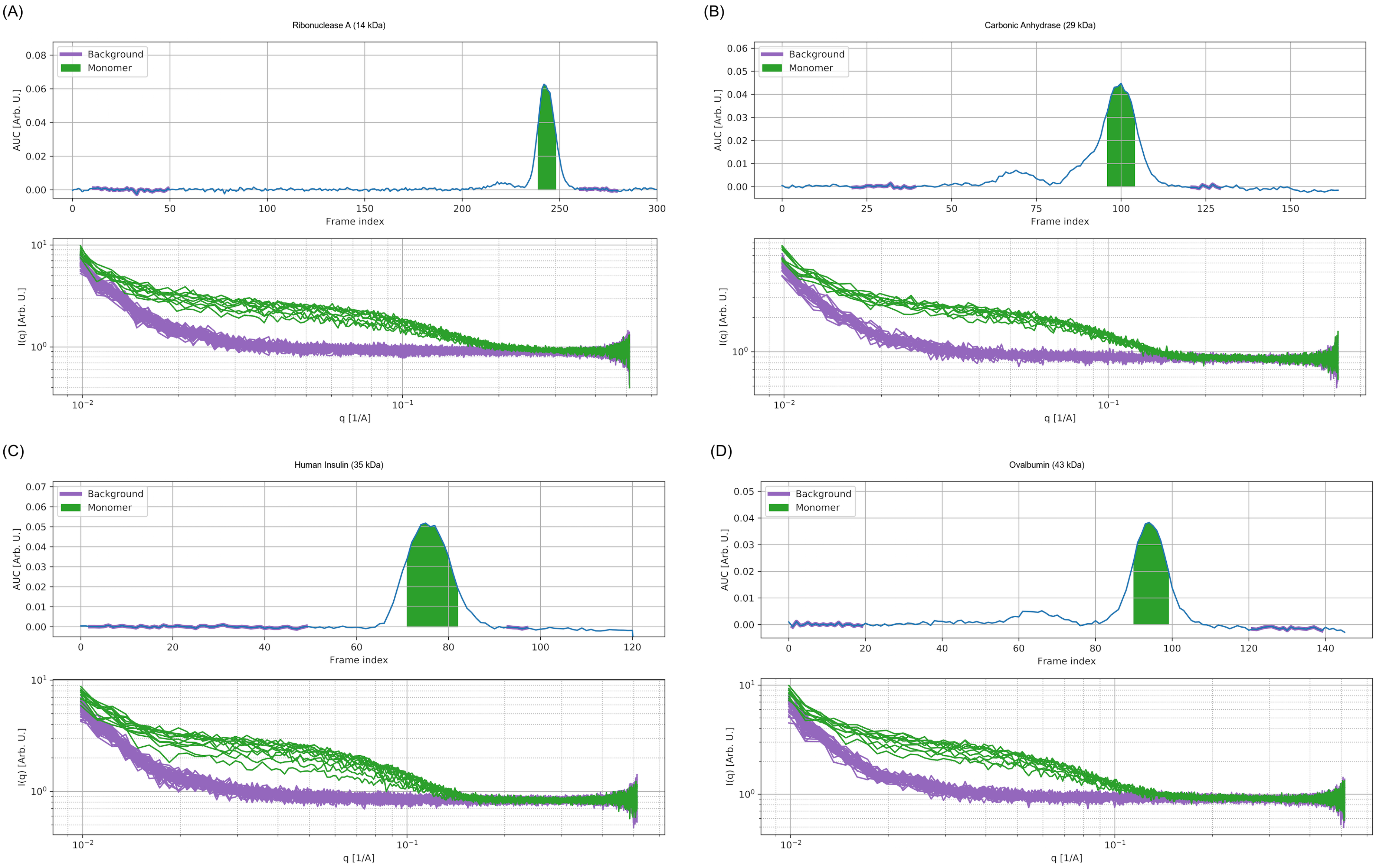
(A)
Ribonuclease A (14 kDa)
Background
Monomer
AUC [Arb. U.]
Frame index
I(q) [Arb. U.]
q [1/A]
(B)
Carbonic Anhydrase (29 kDa)
Background
Monomer
AUC [Arb. U.]
Frame index
I(q) [Arb. U.]
q [1/A]
(C)
Human Insulin (35 kDa)
Background
Monomer
AUC [Arb. U.]
Frame index
I(q) [Arb. U.]
q [1/A]
(D)
Ovalbumin (43 kDa)
Background
Monomer
AUC [Arb. U.]
Frame index
I(q) [Arb. U.]
q [1/A]

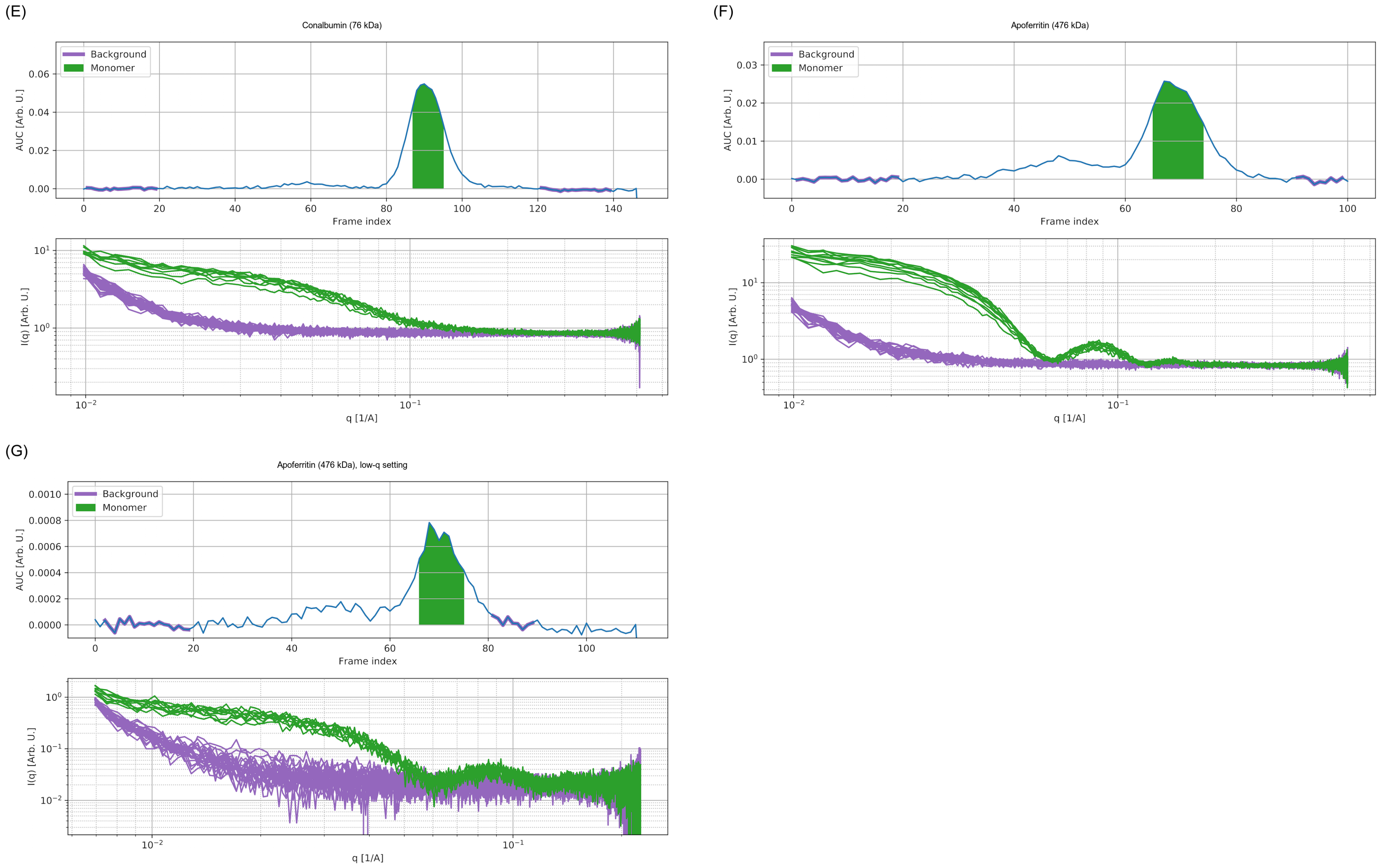

Figure S2: Selection of sample and buffer regions for data analysis of Xenocs BioXolver L SEC-SAXS data. *Top panels:* Integrated intensity as a function of frame index (i.e. time), *bottom panels:* Individual 30s frames.

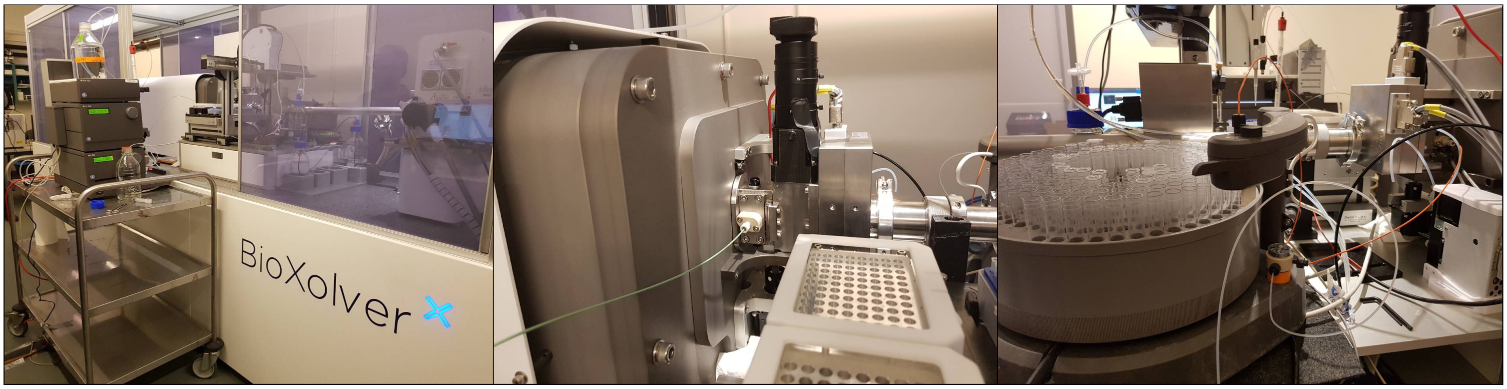

Figure S3: Pictures of our laboratory-based SEC-SAXS setup. Left: Overall view of the mobile HPLC unit next to the SAXS instrument, middle: connection of the HPLC tubing to the flow-through cell, right: fraction collector after the SAXS exposure cell.

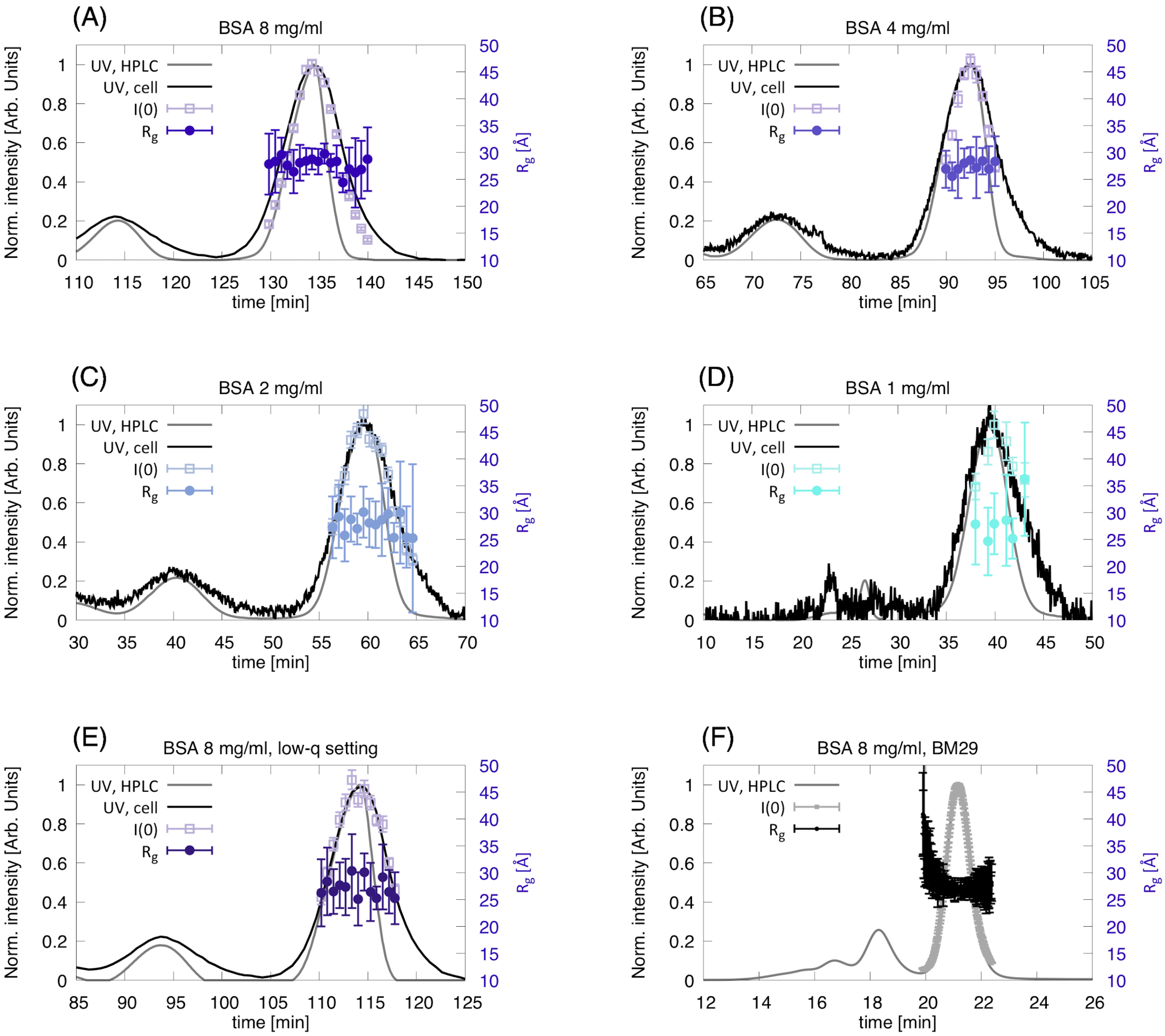

Figure S4: UV traces (HPLC unit and SAXS exposure cell) together with the forward scattering intensity $I(0)$ (left axis) and the radius of gyration R_g (right axis) of each individual frame across the monomer peak of BSA. A-E: Xenocs BioXolver L, F: synchrotron BioSAXS beamline BM29, ESRF-Grenoble.

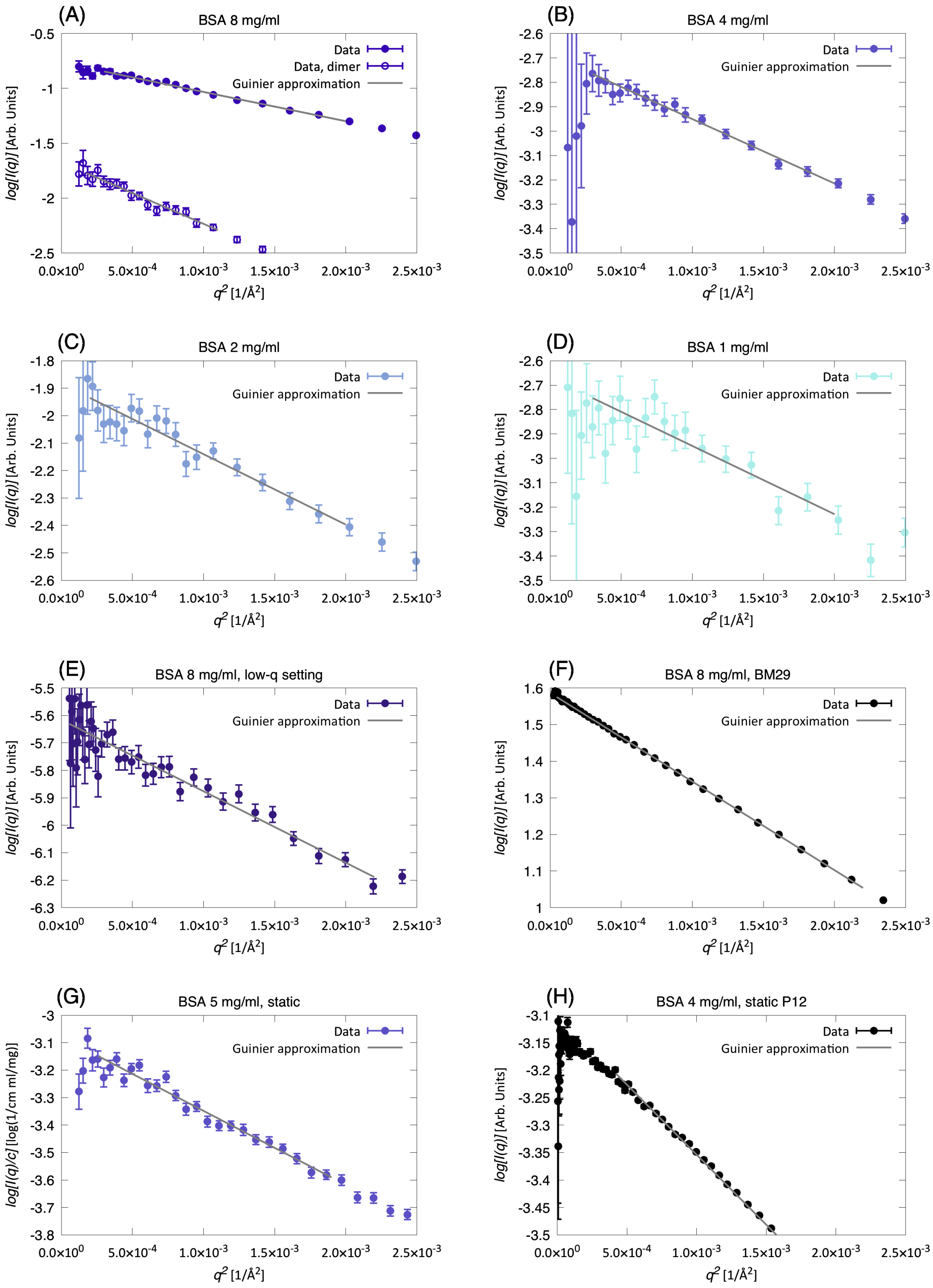

Figure S5: Guinier plots of BSA data. A-F: SEC-SAXS data, G and H: static SAXS data. A-E and G: laboratory instrument (Xenocs BioXolver L), F: synchrotron BioSAXS beamline BM29, ESRF-Grenoble, H: synchrotron BioSAXS beamline P12, EMBL-Hamburg.

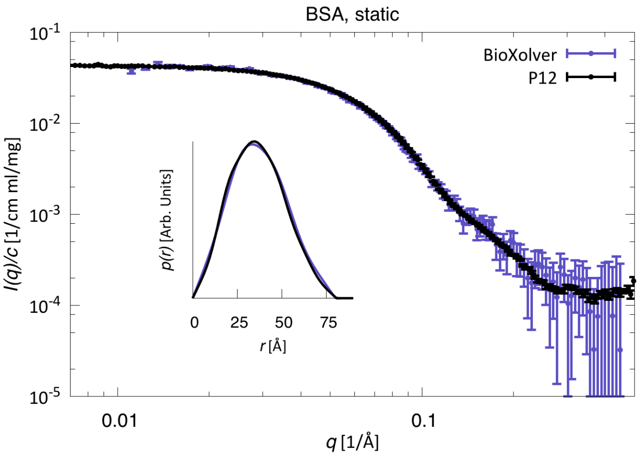

Figure S6: Concentration-normalized static SAXS measurements of 5 mg/ml BSA on our laboratory instrument (Xenocs BioXolver L, 60s exposure) and of 4 mg/ml BSA on a synchrotron BioSAXS beamline (P12, EMBL-Hamburg, 1s exposure) on absolute scale. The synchrotron data was scaled to overlap with the BioXolver data. The inset shows the corresponding pair-distance distribution functions $p(r)$.

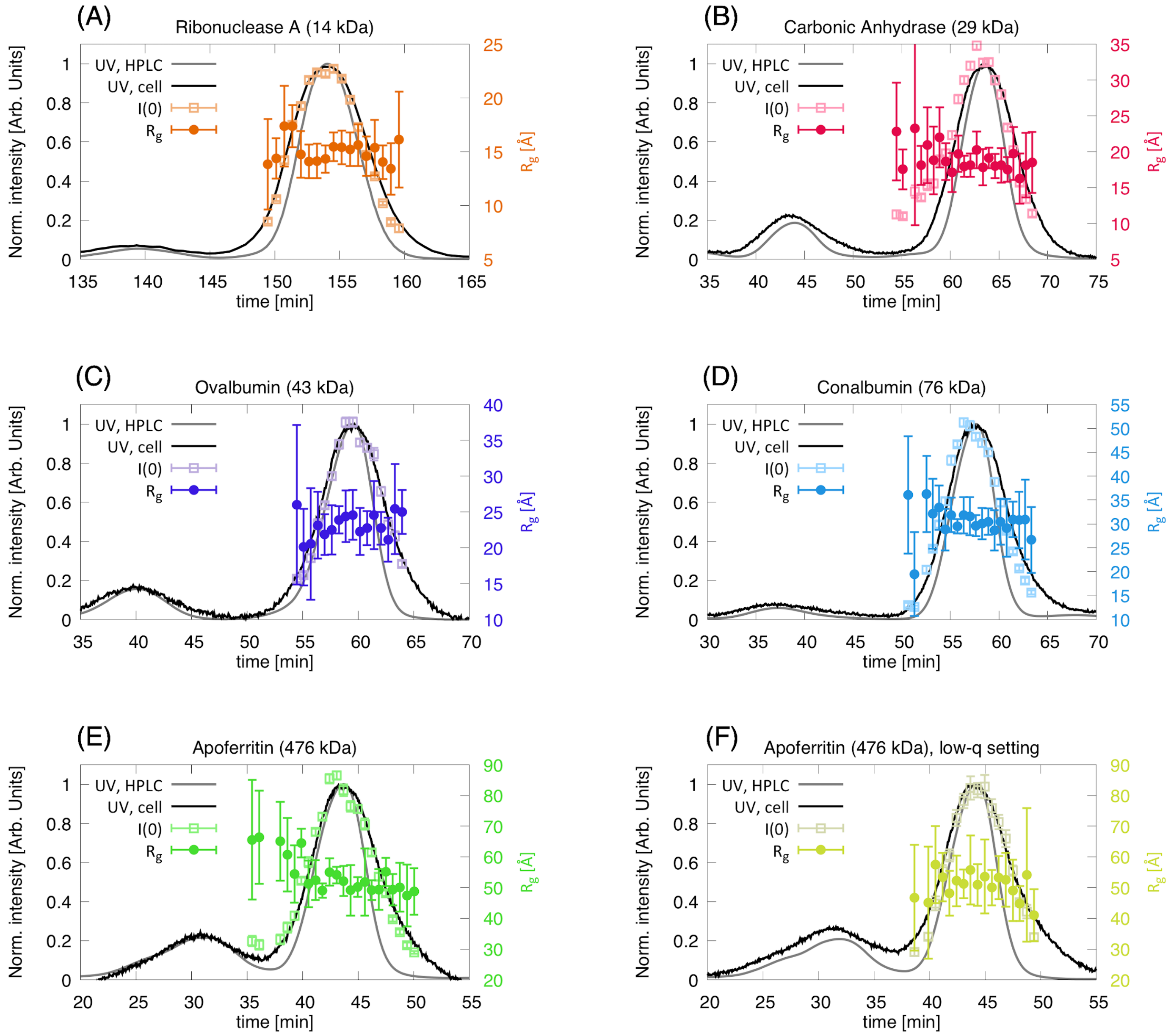

Figure S7: UV traces (HPLC unit and SAXS exposure cell) together with the forward scattering intensity $I(0)$ (left axis) and the radius of gyration R_g (right axis) of each individual frame across the monomer peak of different proteins. *NB:* for HI, no chromatogram is available due to the presence of phenol, which strongly absorbs at 280 nm, in the running buffer.

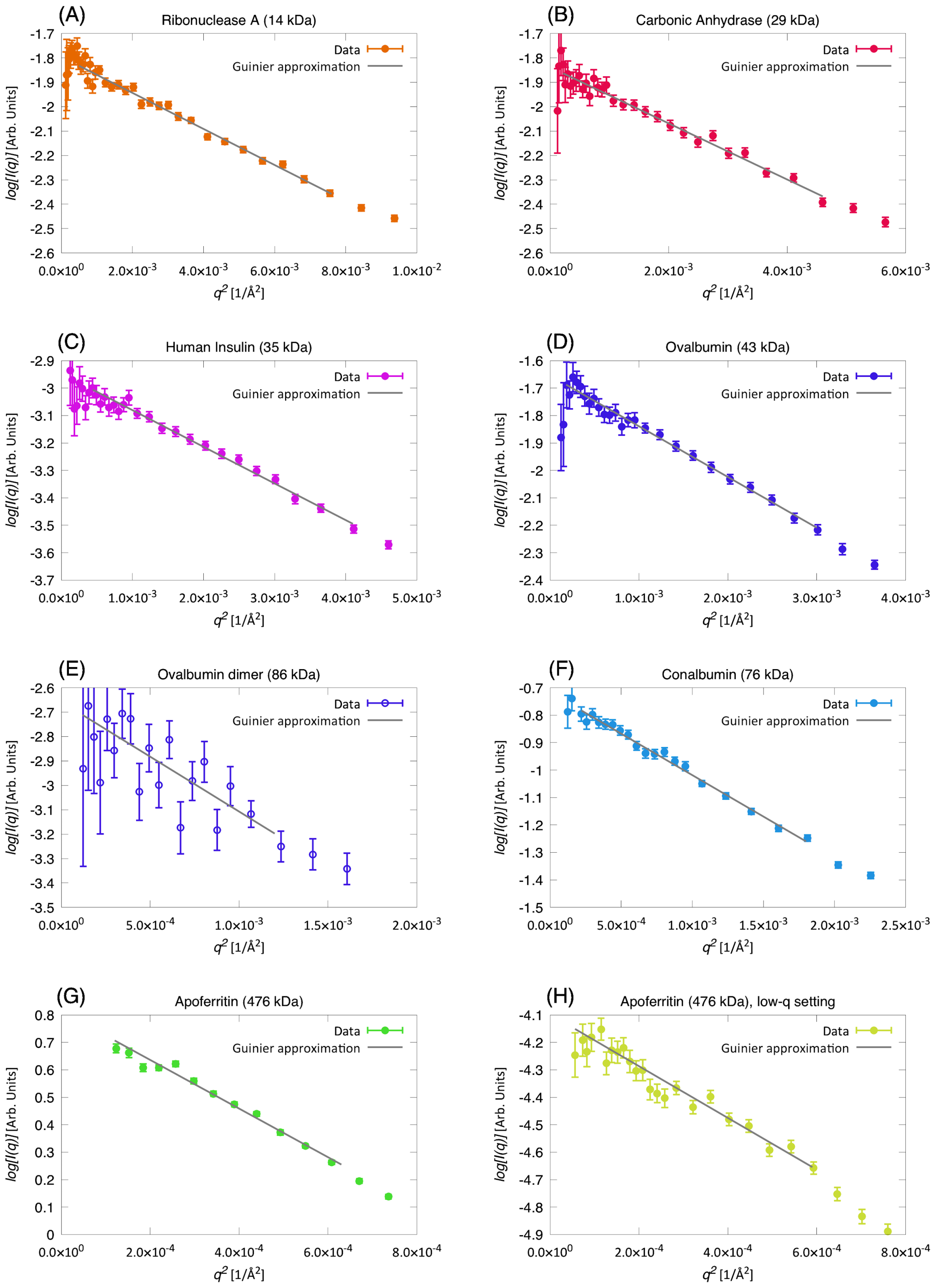

Figure S8: Guinier plots of SEC-SAXS data of ribonuclease A (A), carbonic anhydrase (B), human insulin (C), ovalbumin monomer and dimer (D and E, respectively), conalbumin (F) and apoferritin (G and H).

References

(1) Bujacz, A *Acta Crystallogr. D Biol. Crystallogr.* **2012**, *68*, 1278–1289.

(2) Tilton, R. F.; Dewan, J. C.; Petsko, G. A. *Biochemistry* **1992**, *31*, 2469–2481.

(3) Saito, R; Sato, T; Ikai, A; Tanaka, N *Acta Crystallogr. D Biol. Crystallogr.* **2004**, *60*, 792–795.

(4) Smith, G. D.; Ciszak, E; Magrum, L. A.; Pangborn, W. A.; Blessing, R. H. *Acta Crystallogr. D Biol. Crystallogr.* **2000**, *56*, 1541–1548.

(5) Stein, P. E.; Leslie, A. G.; Finch, J. T.; Carrell, R. W. *J. Mol. Biol.* **1991**, *221*, 941–959.

(6) Kurokawa, H.; Dewan, J. C.; Mikami, B.; Sacchettini, J. C.; Hirose, M. *J. Biol. Chem.* **1999**, *274*, 28445–28452.

(7) Granier, T.; Gallois, B.; Dautant, A.; Langlois d'Estaintot, B.; Précigoux, G. *Acta Crystallogr D Biol Crystallogr.* **1997**, *53*, 580–587.

(8) Nielsen, S.; Toft, K.; Snakenborg, D.; Jeppesen, M.; Jacobsen, J.; Vestergaard, B.; Kutter, J.; Arleth, L. *J. Appl. Crystallogr.* **2009**, *42*, 959–964.

(9) Hopkins, J.; Gillilan, R.; Skou, S *J. Appl. Crystallogr.* **2017**, *50*, 1545–1553.

(10) Konarev, P.; Svergun, D. *IUCrJ* **2015**, *2*, 352–60.

(11) Petoukhov, M. V.; Konarev, P. V.; Kikhney, A. G.; Svergun, D. I. *J. Appl. Crystallogr.* **2007**, *40*, s223–s228.

(12) Vestergaard, B.; Hansen, S. *J. Appl. Crystallogr.* **2006**, *39*, 797–804.

(13) Hansen, S. *J. Appl. Crystallogr.* **2012**, *45*, 566–567.

(14) Fischer, H.; Neto, M.; Napolitano, H.; Polikarpov, I.; Craievich, A. *J. Appl. Crystallogr.* **2010**, *43*, 101–109.

(15) Franke, D; Svergun, D. *J. Appl. Crystallogr.* **2009**, *42*, 342–346.

(16) Volkov, V. V.; Svergun, D. *J. Appl. Crystallogr.* **2003**, *36*, 860–864.

(17) Svergun, D; Barberato, C.; Koch, M. H. *J. Appl. Crystallogr.* **1995**, *28*, 768–773.

(18) Midtgaard, S.; Darwish, T.; Pedersen, M.; Huda, P.; Larsen, A.; Jensen, G.; Kynde, S.; Nicholas, S.; Nielsen, A.; Olesen, C.; Blaise, M.; Dorosz, J.; Thorsen, T.; Venskutonytė, R.; Krintel, C.; Møller, J.; Frielinghaus, H.; Gilbert, E.; Martel, A.; Kastrup, J.; Jensen, P.; Nissen, P.; Arleth, L. *FEBS J.* **2018**, *285*, 357–371.

(19) Nygaard, J.; Munch, H. K.; Thulstrup, P. W.; Christensen, N. J.; Thomas, H.; Jensen, K. J.; Arleth, L. *Langmuir* **2012**, *28*, 12159–12170.

(20) Orthaber, D; Bergmann, A; Glatter, O *J. Appl. Crystallogr.* **2000**, *33*, 218–225.
